# Oxidized Lipoprotein as a Major Vessel Cell Proliferator in Oxidized Human Serum

**DOI:** 10.1371/journal.pone.0160530

**Published:** 2016-08-02

**Authors:** Yoshiro Saito, Noriko Noguchi

**Affiliations:** Department of Medical Life Systems, Faculty of Life and Medical Sciences, Doshisha University, Kyotanabe, Kyoto 610–0321, Japan; Hokkaido Daigaku, JAPAN

## Abstract

Oxidative stress is correlated with the incidence of several diseases such as atherosclerosis and cancer, and oxidized biomolecules have been determined as biomarkers of oxidative stress; however, the detailed molecular relationship between generated oxidation products and the promotion of diseases has not been fully elucidated. In the present study, to clarify the role of serum oxidation products in vessel cell proliferation, which is related to the incidence of atherosclerosis and cancer, the major vessel cell proliferator in oxidized human serum was investigated. Oxidized human serum was prepared by free radical exposure, separated using gel chromatography, and then each fraction was added to several kinds of vessel cells including endothelial cells and smooth muscle cells. It was found that a high molecular weight fraction in oxidized human serum specifically induced vessel cell proliferation. Oxidized lipids were contained in this high molecular weight fraction, while cell proliferation activity was not observed in oxidized lipoprotein-deficient serum. Oxidized low-density lipoproteins induced vessel cell proliferation in a concentration-dependent manner. Taken together, these results indicate that oxidized lipoproteins containing lipid oxidation products function as a major vessel cell proliferator in oxidized human serum. These findings strongly indicate the relevance of determination of oxidized lipoproteins and lipid oxidation products in the diagnosis of vessel cell proliferation-related diseases such as atherosclerosis and cancer.

## Introduction

Free radicals and oxidative stress are involved in a variety of pathological events such as atherosclerosis, cancer, ischemia–reperfusion, and neurodegenerative diseases [1–3]. The oxidation of biological molecules by free radicals yields a variety of oxidation products. Oxidation of lipids and proteins has been the subject of extensive studies for several decades, and its mechanisms, dynamics, and products have been investigated [4, 5]. The oxidation of biological materials may induce the loss of fine structure and natural function, while it could give novel biological activity, which play an important role as regulatory mediators in signaling processes [6, 7]. It is known that polyunsaturated fatty acids (PUFAs) and their esters are vulnerable to oxidation and that their susceptibility to oxidation increases with an increase in the number of double bonds [8]. Lipid peroxidation initiated by free radical exposure in human plasma results in the formation of oxidized lipoproteins including oxidized low-density lipoprotein (oxLDL), and cholesteryl ester hydroperoxide (CE-OOH) is generated as a major lipid peroxidation product [9]. Protein oxidation by free radicals could generate protein carbonyl derivatives [10]. These oxidized products are measured as biomarkers of oxidative stress to assess the oxidative injury in the pathologic processes of free radical-related diseases.

The proliferation of vascular cells is related to the onset as well as the progress of several diseases such as atherosclerosis and cancer [11–13]. In atherosclerosis, the proliferation and migration of vascular smooth muscle cells (VSMCs) are the pivotal events of atherogenesis and play an essential role in atherosclerotic plaque progression [11, 14]. Proliferative VSMCs result in the development of neointimal hyperplasia, which is implicated in coronary restenosis after angioplasty in patients with coronary heart disease [14]. The proliferative activity of VSMCs is regulated by many growth promoters and inflammatory factors, such as platelet-derived growth factor, endothelin-1, angiotensin II, and oxLDL [14–16]. On the other hand, in the case of cancer, blood vessels supply oxygen and nutrients to tumors and help them to become large [12, 13, 17]. Tumors secrete proangiogenic growth factors, such as vascular endothelial growth factor (VEGF), which activate angiogenic signaling to induce the proliferation of endothelial cells (ECs). ECs face the blood vessel lumen and form a single layer, the endothelium, which controls vessel function. ECs in tumors are highly activated and show hyperproliferation, which greatly contributes to tumor development [17, 18].

To understand the relationship between oxidative stress and the pathology of several diseases, biomarkers of oxidative stress such as oxidized lipids, proteins, and DNA have been evaluated; however, the relationship between oxidative stress biomarkers and their biological action has not been well investigated. In the present study, using human VSMCs and ECs, the biological activity of free radical-treated human serum was examined, and a major proliferator of vascular cells in oxidized human serum was investigated.

## Results

### Preparation of oxidized human serum and determination of oxidation products

To examine the effects of oxidized products in serum, a water-soluble radical initiator, 2,2-azobis[2-(2-imidazolin-2 yl)propane]dihydrochloride (AIPH), at 5 mM was added to 50% human serum in PBS, and the serum component was oxidized for 8 h at 37°C, as described previously [9]. AIPH decomposes thermally to give free carbon-centered radicals, which react with oxygen rapidly to yield peroxyl radicals [19]. Oxidation of serum components was confirmed by lipid and protein oxidation products. Determinants are summarized in Table 1. CE-OOH, a major lipid peroxidation product in peroxyl radical-treated serum, increased from 1.1 to 192 μM by AIPH treatment. A high concentration of CE-OOH in human serum before oxidation was detected at 1.1 μM, suggesting the increased levels of CE-OOH during the preparation of human serum and storage; however, AIPH treatment resulted in an obvious increase in CE-OOH (Table 1). The increase in protein oxidation products was confirmed by the evaluation of protein carbonyl, which increased from 39 to 148 μM. Prepared oxidized human serum was separated by gel chromatography, and each fraction was used for further experiments.

**Table 1:** Contents of lipid peroxidation products, protein carbonyl, and lipids in human serum samples used in this study^a^.

	CE-OOH	CE-OH	CE-O(O)H	Carbonyl	FC	CE (20:4)	CE (18:2)	CE (16:0)
Human serum	1.06	< 0.28	1.06	39.1	788	197	760	438
Lipid fraction (L)	< 0.28	< 0.28	< 0.28	nd	1330	411	1510	656
Oxidized HS	192	60.8	253	148	922	222	1040	564
Oxidized LPDS	4.54	2.86	7.40	216	45.6	1.32	16.7	7.26
Oxidized LPDS + L	159	38.2	197	nd	706	123	628	330

(μM)

^a^ Contents of lipid peroxidation products, protein carbonyl, and lipids in human serum samples were measured as described in Materials and Methods. The mean values of each content in the serum from the same subject are shown (n = 3). L: Lipid fraction, HS: human serum, LPDS: lipoprotein-deficient serum, LPDS + L: Lipid fractions were added to LPDS, nd: not done

### Effects of oxidized human serum components on the viability of vessel cells

**Fig 1:**
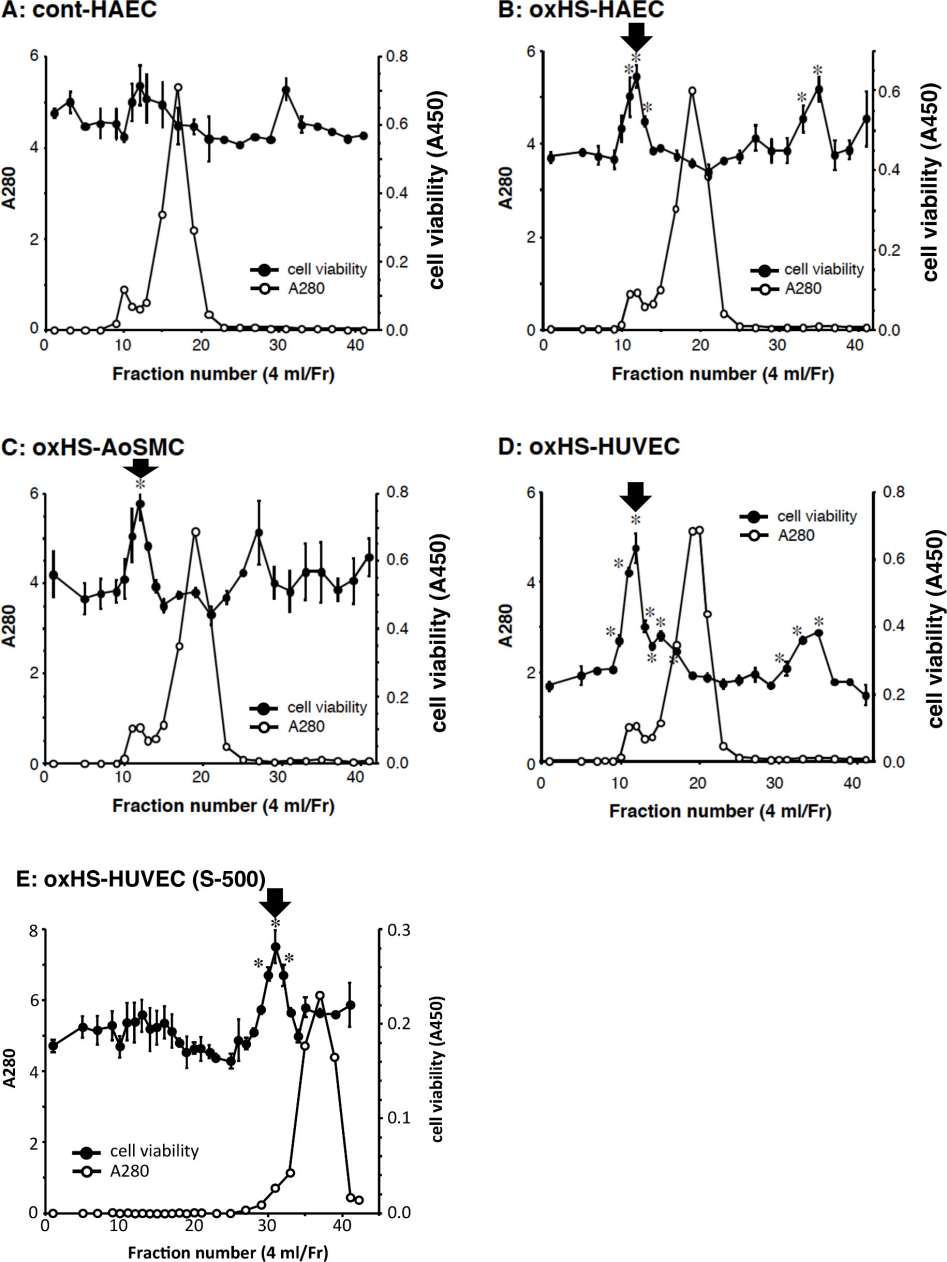
Effects of oxidized human serum components on vessel cell viability. **A-D.** Control (**A**) and oxidized human serum (**B-D**) were fractionated by Sephacryl S-300 gel chromatography, and protein contents were determined by absorbance at 280 nm. HAEC (**A, B**), AoSMC (**C**), and HUVEC (**D**) were treated with each fraction (10%) for 48h, and the viability was measured by WST assay, as described under “Materials and methods”. The maclomolecule proliferator for vessel cells at Fr.12 is indicated by black arrow. **E.** Oxidized human serum were fractionated by Sephacryl S-500 gel chromatography, and HUVEC was treated each fraction for 48h. The maclomolecule proliferator for vessel cells at Fr.31 is indicated by black arrow. * *P* < 0.01, compared with vehicle control.

Oxidized human serum was applied to Sephacryl S-300 gel chromatography, and then each fraction was added to the culture media of vessel cells such as ECs and SMCs. After 48 h, cell viability was determined. Cell viability and serum protein contents in each fraction are plotted in Fig 1. In the case of human aortic endothelial cells (HAECs), no statistically significant change was observed in the component of control human serum (Fig 1A). In contrast, a significant increase in HAEC viability was observed in the case of oxidized human serum, in which two peaks are observed: one is a macromolecule of more than 200 kDa at fraction number 12 (Fr. 12) and the other is of low molecular weight at Fr. 36 (Fig 1B). In the other types of vessel cells such as human aortic smooth muscle cells (AoSMCs) and human umbilical vein endothelial cells (HUVECs), a statistically significant increase was observed in macromolecule peaks at Fr. 12 (Fig 1C and 1D). To confirm the precise molecular weight of the macromolecule proliferator in oxidized human serum with a single peak, Sephacryl S-500 gel chromatography, with a larger pore size than that of Sephacryl S-300, was conducted; a significant increase in vessel cell viability with a single peak at around 200 kDa was observed (Fig 1E). These results suggest that the macromolecule proliferator for vessel cells is generated in oxidized human serum.

### Distribution of oxidized products in gel chromatography

**Fig 2:**
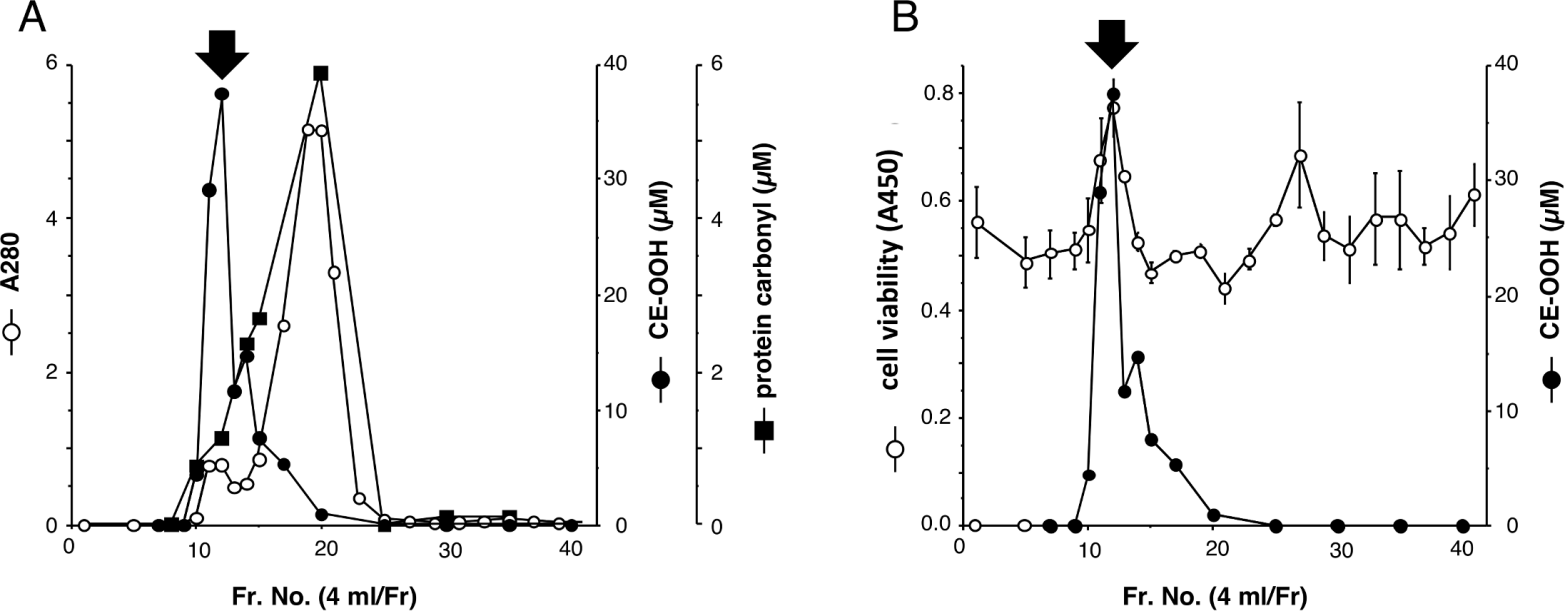
Distribution of oxidized products in Sephacryl S-300 gel chromatography. **A.** Oxidized human serum were fractionated by Sephacryl S-300 gel chromatography, and contents of protein (A280), cholesterol ester hydroperoxide (CE-OOH), and protein carbonyl were determined, respectively. **B.** Both AoSMC viability and CE-OOH contents are plotted. The peak of maclomolecule proliferator for vessel cells and CE-OOH at Fr.12 is indicated by black arrow.

The distribution of oxidized products, such as CE-OOH and protein carbonyls, in gel chromatography of human oxidized serum was analyzed. The peak of CE-OOH was observed at Fr. 12, while the peak of protein carbonyls was found at Fr. 20 and its distribution was similar to that of protein content determined by absorbance at 280 nm (Fig 2A). The peak of CE-OOH was consistent with that of the macromolecular proliferator for vessel cells in oxidized human serum (Fig 2B). CE-OOH mainly exists in lipoproteins. Thus, these results suggest that oxidized lipoproteins containing CE-OOH act as the macromolecular proliferator for vessel cells in oxidized human serum.

### Effects of oxidized lipoprotein-deficient serum on the viability of vessel cells

**Fig 3:**
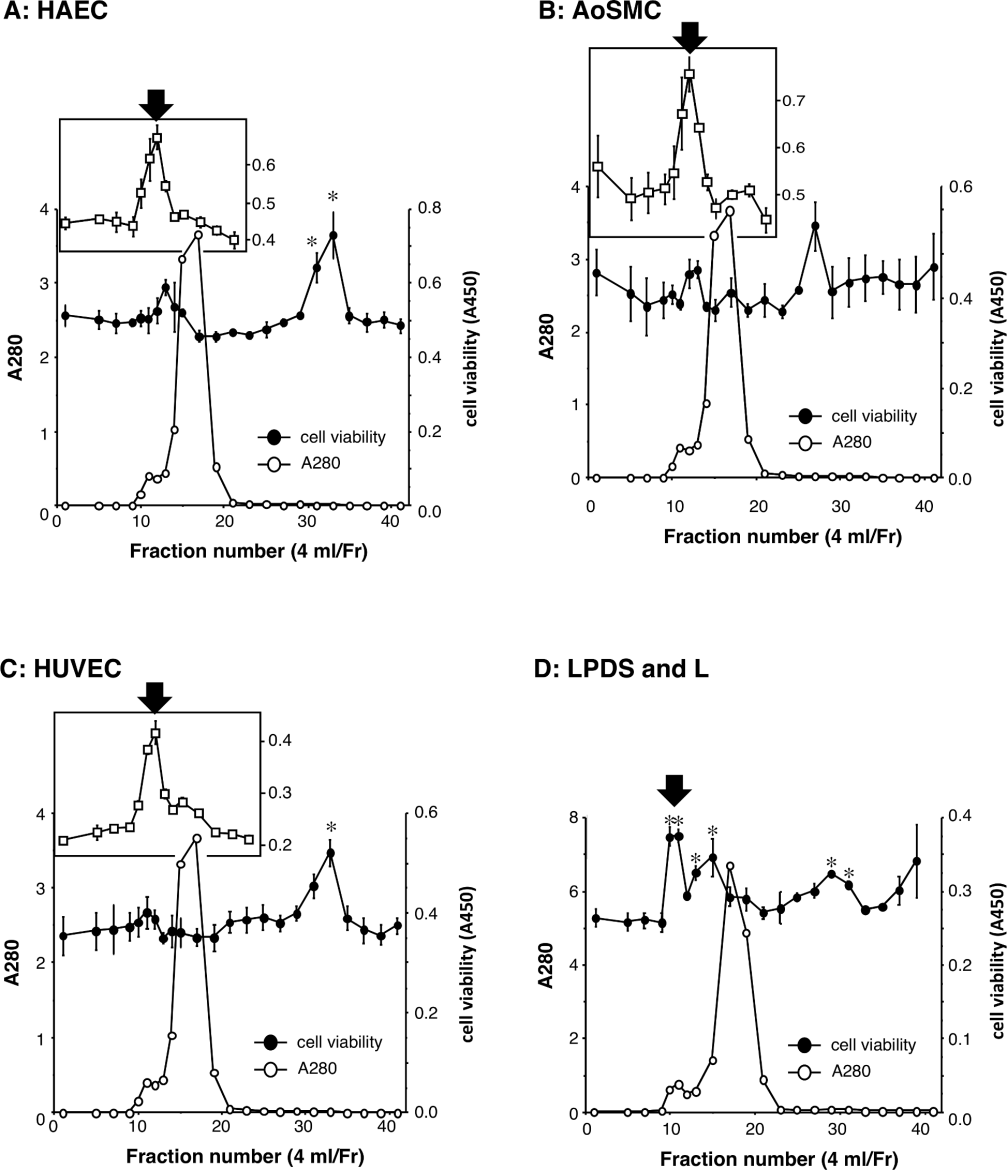
Effects of oxidized lipoprotein deficient serum on the viability of vessel cells. **A-C.** Oxidized lipoprotein deficient serum (LPDS) were fractionated by Sephacryl S-300 gel chromatography, and protein contents were determined by absorbance at 280 nm. HAEC (**A**), AoSMC (**B**), and HUVEC (**C**) were treated with each fraction (10%) for 48h, and the viability was measured by WST assay. The cell viability in the case of oxidized human serum fractions are shown in inner figure. **D.** LPDS and extracted lipid fraction (L) were mixed, oxidized, and then fractionated by Sephacryl S-300 gel chromatography. HAEC was treated with each fraction (10%) for 48h, and the viability was measured. The maclomolecule proliferator for vessel cells at Fr.12 is indicated by black arrow. * *P* < 0.01, compared with vehicle control.

To investigate the involvement of oxidized lipoproteins, lipoprotein-deficient serum (LPDS) was prepared, and both FC and CE were detected in lipid fraction extracted from human serum. LPDS was oxidized by peroxyl radical initiator AIPH. The disappearance of lipids as well as oxidized lipids was confirmed in oxidized LPDS, while high levels of protein carbonyl were detected (Table 1). Oxidized LPDS was applied to Sephacryl S-300 gel chromatography, and then each fraction was added to the culture media of vessel cells. It was found that the significant increase in cell viability of HAECs disappeared in a high molecular weight fraction of oxidized LPDS at Fr. 12 (Fig 3A). In contrast, HAEC viability increased at Fr. 12 when cultured with oxidized human serum components (Fig 3A inset). In other types of cells such as AoSMCs and HUVECs, a significant increase in cell viability at Fr. 12 disappeared in the case of oxidized LPDS (Fig 3B and 3C), although a high molecular weight fraction of oxidized human serum increased the viability of these cells (Fig 3B and 3C inset). Collectively, these results suggest that the activity of the macromolecular proliferator for vessel cells in oxidized human serum is derived from oxidized lipoproteins.

To investigate the involvement of oxidized lipoproteins, extracted lipids (L) were put back into LPDS and then oxidized. The content of CE-OOH in oxidized LPDS with L was confirmed, which was slightly lower than that of oxidized human serum because of the loss of lipoproteins during the isolation procedure (Table 1). Oxidized LPDS with L was separated by gel chromatography and each fraction was added to the cells. As a result, the significant increase in cell viability at Fr. 12 was recovered in oxidized LPDS with L (Fig 3D).

### Effects of oxidized low-density lipoprotein on the viability of vessel cells

**Fig 4:**
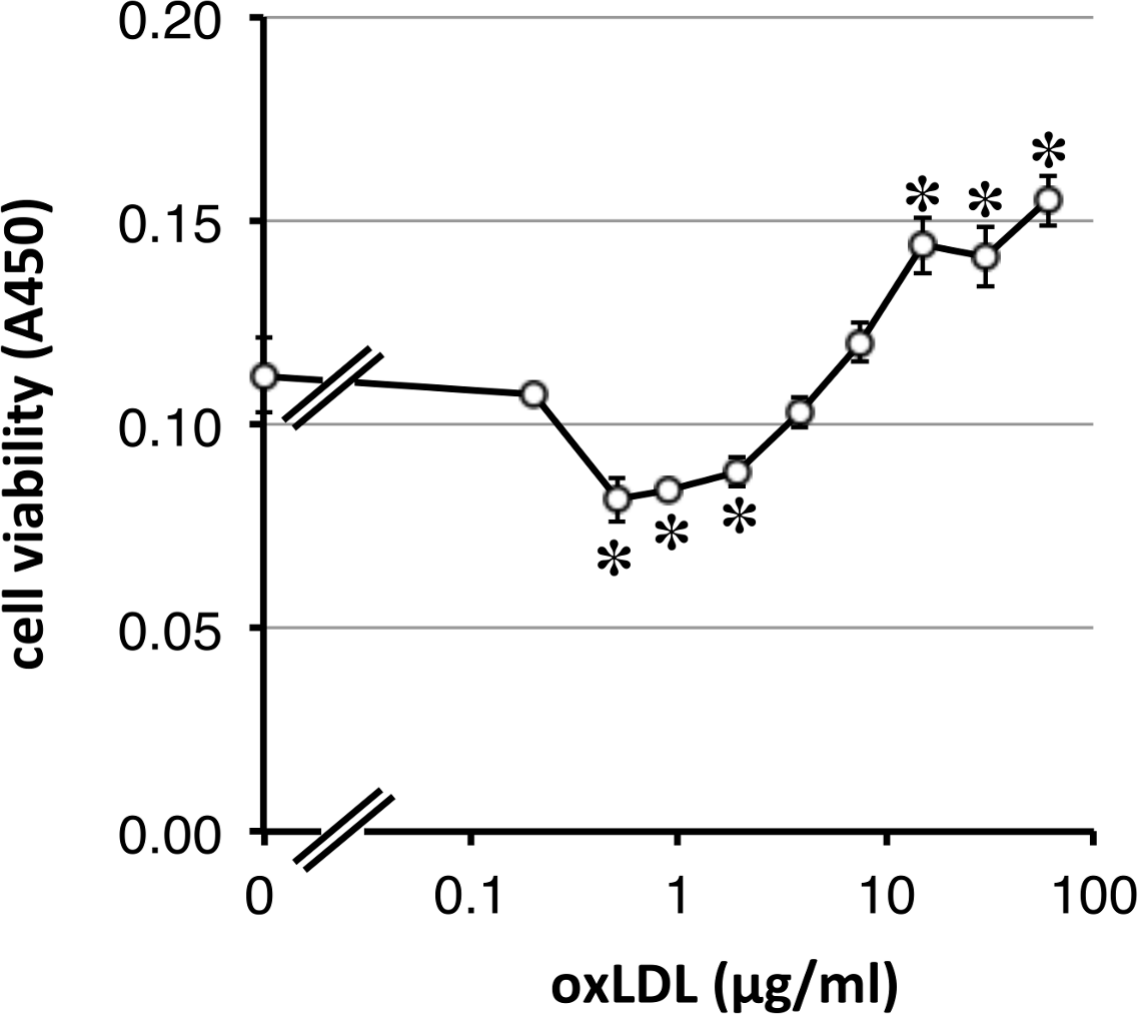
Effects of oxidized low density lipoprotein on the viability of vessel cells. HUVEC was treated with variable concentrations of oxidized LDL for 48h, and the viability was measured by WST assay. * *P* < 0.01, compared with vehicle control.

The effects of oxLDL on the viability of vessel cells were further examined. Purified LDL was oxidized by peroxyl radical initiator AIPH and the radical initiator was removed by dialysis to PBS. Different concentrations of oxLDL were added and cell viability was determined. Cell viability changed in a concentration-dependent manner; it significantly decreased at lower concentrations around 1 μg/ml, while it significantly increased at concentrations of more than 20 μg/ml (Fig 4). These results suggest that oxidized lipoproteins could act as the proliferator for vessel cells concentration-dependent manner.

## Discussion

In the present study using oxidized human serum, we could identify oxidized lipoproteins, containing CE-OOH, as a major proliferator of vascular cells. This result clearly suggests the relationship between oxidative stress and the proliferation of vessel cells, which are related to the progression of arteriosclerosis and cancer, and suggests the significance of oxidized lipoproteins and lipids in the evaluation of the pathogenesis of atherosclerosis and cancer.

Lipid peroxidation products in serum have been well characterized, and the increase in oxidized products of CE has been reported in free radical-induced serum lipid peroxidation [19–21]. Lipid peroxidation products in plasma of atherosclerosis are higher than those in other diseases [22]. The accumulation of oxidized CEs in human atherosclerotic tissue has also been reported [23, 24]. Thus, these lines of evidences strongly indicate the occurrence of lipid peroxidation in atherosclerosis. In the present study, we used a water-soluble radical initiator AIPH to achieve oxidation of biological molecules. This azo compound could generate free radicals at a constant rate for a specific duration, resulting in a highly reproducible oxidation of human serum [19]. However, there are considerable differences between oxidized lipoproteins prepared by AIPH and oxidized lipoproteins in patients with atherosclerosis [25]. It is important to elucidate whether oxidized lipoproteins in the serum of atherosclerosis patients could be identified as a major vessel cell proliferator.

In the present study, we confirmed that the amount of CE linoleate (18:2) was higher than that of CE arachidonate (20:4) and CE palmitate (16:0) (Table 1). Although phospholipids such as phosphatidylcholines are good targets of free radical-induced lipid peroxidation, the amount of CE is higher than that of phospholipids, and oxidized products of CE are detected as major lipid oxidation products in plasma treated with free radicals [9, 19]. Free radicals oxidize several biomolecules such as lipids and proteins in lipoproteins. It is noteworthy that the contents of protein carbonyl (39 μM) were higher than those of CE-O(O)H (1 μM) in unoxidized human serum, while a lower protein carbonyl content (148 μM) than CE-O(O)H (253 μM) was observed in oxidized human serum. The major protein carbonyl in oxidized human serum was detected at Fr. 20 (Fig 2A); however, a significant increase in the viability of vascular cells was not observed in this fraction. These results suggest that CEs in lipoproteins are good targets of free radical-mediated oxidation and that oxidized lipoproteins containing CE-O(O)H act as a proliferator of vessel cells. Furthermore, our results suggest that the evaluation of CE-O(O)H is important in atherosclerosis and cancer to assume the effects of oxidative stress and the quality of vascular cells. However, this does not obviate the need to take into account other lipid classes affecting vascular cells in atherosclerosis and cancer.

Treatment of human serum with free radical initiator increased the contents of CE-OOH approximately 200-fold to 192 μM (Table 1), and around 40 μM of CE-OOH was determined at the peak of a major proliferator of vascular cells, Fr. 12 (Fig 2A). In the present study, each fraction was added at a final concentration of 10%; therefore, vascular cells were exposed to oxidized lipoproteins with around 4 μM CE-OOH. Oxidized LDL at a final concentration of 20–60 μg/ml, which contained 6–18 μM CE-OOH, significantly increased its cell viability (Fig 4). Therefore, it appears that oxidized lipoproteins with micromolar levels of CE-OOH could induce vascular cell proliferation. In the case of oxidized LPDS + L, it is thought that oxidized lipoproteins induced significant cell proliferation (Fig 3D). Since other oxidized lipoproteins such as oxidized high-density lipoprotein (HDL) have also been reported to induce vascular cell proliferation [26], the role of oxidized lipoproteins as a vessel cell proliferator might not be restricted to oxidized LDL. To investigate the involvement of oxidative stress on the proliferation of vascular cells, hydrogen peroxide and cumene hydroperoxide were added and cell viability was determined; however, a siginificant increase in cell viability was not observed with either stressor (Supporting information S1 and S2 Figs). It has been reported that oxidized lipids in oxidized lipoproteins are incorporated into the cells and that oxidized lipids such as CE-OOH are detected in cell extracts [27]. Oxidized lipoproteins are known to bind several receptors including lectin-type oxidized LDL receptor 1 (LOX-1), which is related to vessel cell proliferation [28]. Incorporated oxidized lipids might relate to the proliferation of vascular cells; however, the precise molecular mechanisms of proliferation of vascular cells induced by oxidized lipoproteins, particularly the role of CE-O(O)H, have not been fully elucidated. It is therefore imortant to identify the proliferator in oxidized lipoproteins.

In conclusion, this study could identify oxidized lipoproteins as a major proliferator of vascular cells in human serum treated with radical initiator, and indicates the significance for the evaluation of oxidized lipoproteins and lipids in atherosclerosis and cancer patients to assume disorder of vessel cells.

## Materials and Methods

### Preparation of oxidized human serum

Experimental procedures including fractionation of human plasma were approved by the Ethics Committee of Doshisha University. The participant provided written informed consent to participate in this study. After an overnight fast, blood from a healthy volunteer was collected in tubes. Serum was obtained by centrifugation at 3,000 *g* for 5 min at 4°C and was stored at -80°C. The oxidation of serum (1/1 with PBS) was carried out at 37°C in PBS solution. The oxidation was initiated by an addition of AIPH (Wako Pure Chemical Industries) at 5 mM for 8 h, as described previously [19].

The contents of CE-OOH, cholesteryl ester hydroxides (CE-OH), free cholesterol (FC), and CE isoforms such as CE (20:4), (18:2), and (16:0) were determined with an HPLC by spectrophotometric detector (SPD-10AV, Shimadzu, Japan) as described previously [27]. The mixture of oxidized samples was extracted with chloroform/methanol (2/1) by twice as volume as the sample and chloroform layer was injected to an HPLC for lipid hydroperoxides analyses. ODS column (LC-18, 5mm, 250 x 4.6mm, Supelco, Japan) was used and acetonitrile/isopropyl alcohol/water (44:54:2 by volume) was eluted at 1 ml/min.

Protein carbonyl content was determined by using colorimetric assay as described previously [29]. Briefly, 10 mg serum protein in 250 μl PBS was reacted with 1 ml of 10 mM dinitrophenylhydrazine (DNP, WAKO) in 2 M HCl for 45 min, and then 1 ml of 28% trichloroacetic acid (TCA) added. TCA precipitants were washed three times with 2.5 ml ethanol/ethyl acetate (1:1). Pellets were dissolved in 1 ml of 6 M guanidine hydrochloride, 0.5 M potassium phosphate, pH 2.5, and absorbance at 375 nm was measured. Carbonyl content was determined as nmol/mg protein using ε_375_ 22,000 M^-1^cm^-1^.

### Gel chromatography

AKTAprime plus (GE Healthcare) was used for the fractionation of serum proteins. Serum samples were applied to a HiPrep 16/60 Sephacryl Columns S-300 HR or S-500 HR (GE Healthcare). Tris-buffered saline (TBS, 20 mM Tris–HCl, pH7.4, containing 150 mM NaCl) was eluted at 0.8 ml/min and fractionated samples were corrected at 4 ml/Fr. Each fractions were stored at -20°C.

### Cell culture and measurement of cell viability

HAECs, AoSMCs, and HUVECs were purchased from LONZA. HAEC and HUVEC were cultured in EGM-2 BulletKit (LONZA), while AoSMC was cultured in SmGM-2 BulletKit (LONZA), containing growth factors with 2% fetal bovine serum, at 37°C in a 5% CO_2_ atmosphere. To examine the effects of oxidized human serum components, cells were grown on 48 well plates at a density of 4 x 10^4^ cells/ml (333 μl/well). After the cells were attached (16–18 h), they were treated with oxidized human serum components at 10% (33 μl/well) for 48 h. To determine cell viability, Premix WST-1 Cell Proliferation Assay System (TAKARA Bio Inc) was used. The cells were incubated with 10% WST-1 at 37°C for 2 h. The optical density of formazan was measured at 450 nm using a Multiskan Ascent plate reader (Thermo Electron).

### Preparation and oxidation of lipoprotein deficient serum and low density lipoprotein

Human LPDS was prepared by ultra-centrifugation. Briefly, a density of human serum was increased to 1.21 g/ml by the addition of KBr, and then lipid fraction (L) and LPDS were corrected in a upper and lower layer, respectively. Each fraction was dialyzed against PBS. Oxidation of LPDS and LPDS plus L was conducted and then applied to gel chromatography, as described above.

LDL was separated by ultra-centrifugation as described in the literature [30] within a density cutoff of 1.019–1.063 g/ml, and then dialyzed against PBS. The protein concentration of LDL was measured using the bicinchonic acid protein assay reagent (Pierce). Oxidation of LDL (1.0 mg protein/ml) was carried out at 37°C under air for 12 h. Oxidation was initiated by the addition of 1mM AIPH. Oxidized LDL was dialyzed against PBS to remove AIPH from the LDL.

### Statistical analysis

The difference between determinations was statistically analysed with analysis of variance (ANOVA) using Tukey’s test for multiple comparisons. Values of *P* < 0.01 were considered as significant.

## Supporting Information

S1 Fig Effects of hydrogen peroxide on the viability of vessel cells. HUVEC was treated with variable concentrations of hydrogen peroxide (H_2_O_2_) for 48h, and the viability was measured by WST assay. * *P* < 0.01, compared with vehicle control.
(TIFF)

S2 Fig Effects of cumene hydroperoxide on the viability of vessel cells. HUVEC was treated with variable concentrations of cumene hydroperoxide (Cumene-OOH) for 48h, and the viability was measured by WST assay. * *P* < 0.01, compared with vehicle control.
(TIFF)
