# Supplementary figures and images for: Oxidized Lipoprotein as a Major Vessel Cell Proliferator in Oxidized Human Serum
Source: PLoS One. 2016 Aug 2;11(8):e0160530. doi: 10.1371/journal.pone.0160530 (PMC4970716; doi:10.1371/journal.pone.0160530)

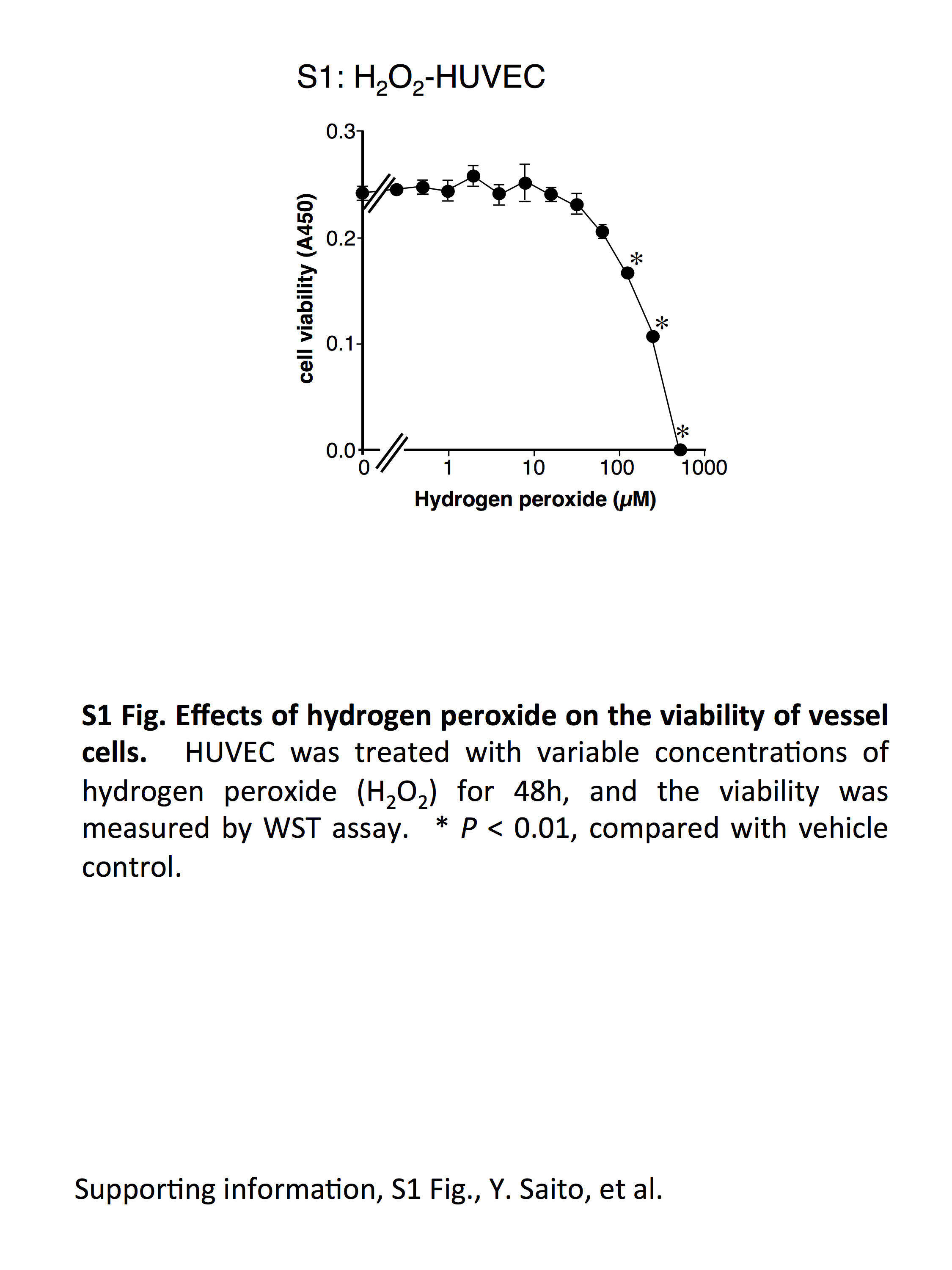

Supplement: S1 Fig — HUVEC was treated with variable concentrations of hydrogen peroxide (H2O2) for 48h, and the viability was measured by WST assay. * P < 0.01, compared with vehicle control. (TIFF) [file pone.0160530.s001.tiff]

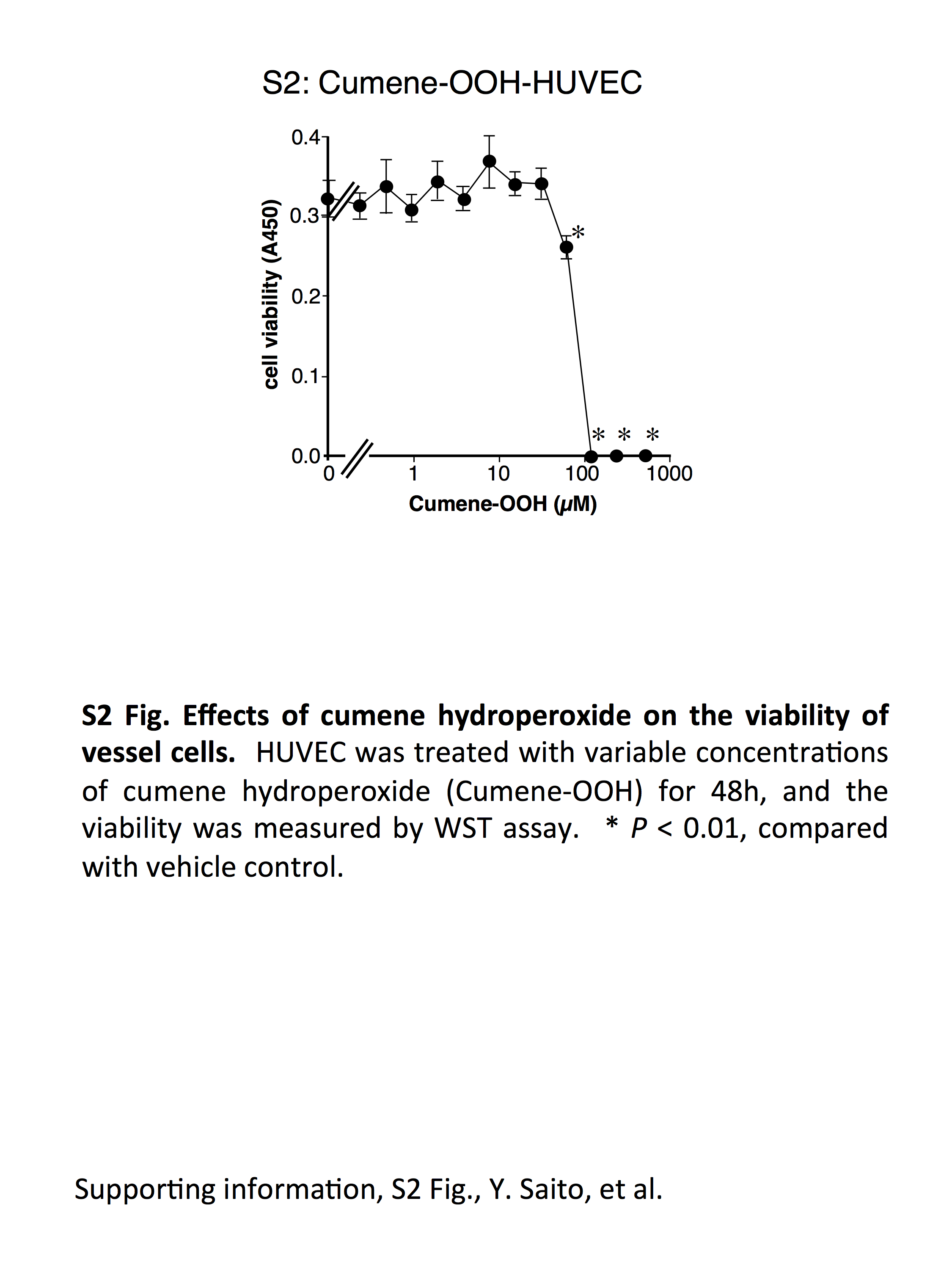

Supplement: S2 Fig — HUVEC was treated with variable concentrations of cumene hydroperoxide (Cumene-OOH) for 48h, and the viability was measured by WST assay. * P < 0.01, compared with vehicle control. (TIFF) [file pone.0160530.s002.tiff]
